# Characterizing the last holdouts

**DOI:** 10.18632/oncotarget.15090

**Published:** 2017-02-04

**Authors:** Erin L. Stewart, Ming-Sound Tsao

**Affiliations:** University Health Network and Princess Margaret Cancer Centre and University of Toronto, Toronto, Ontario, Canada

**Keywords:** adaptive resistance, drug tolerance, EGFR TKIs, metabolomics, lung cancer

Lung cancer is the most common cause of cancer-related death in the world [1]. Tyrosine kinase inhibitors (TKIs) targeting EGFR, have led to multi-fold improvements in survival for the 15-50% of patients with advanced non-small cell lung cancer (NSCLC) carrying activating mutations [1]. However, almost all will acquire resistance and relapse after 1-2 years. The emergence of T790M, a secondary mutation, accounts for approximately 50% of all the resistant mechanisms [2]. Third generation EGFR TKIs that specifically target T790M have been developed and have demonstrated clinical trial response rates of ~60%. Cure for these patients remains unlikely though, as further development of resistance via multiple mechanisms have been observed [2].

The exact origin of acquired resistance to treatments is still debated, with two major theories currently being investigated [2]. One theory is that resistant subclones pre-exist and are selected for by treatment, leading to the development of resistant tumors. The second hypothesis is that a subset of cells, known as drug tolerant cells (DTCs), will adjust sufficiently to survive the therapy, giving them time to acquire mechanisms of resistance [2].

Previously, Fan et al (2011) from Dr. Patrick Ma's group demonstrated that after just 6-9 days of treatment with EGFR TKIs, residual DTCs exhibited increased pSTAT3 and BCL-2/BCL-xL expression [3]. Combination of EGFR TKI with BCL-2/BCL-xL inhibitor (such as navitoclax) significantly hindered the development of DTCs, both *in vitro* and *in vivo*. Others also have identified a potential role for STAT3 in the DTC phenotype, albeit through different mechanisms of activation [4, 5].

The ability of some cancer cells to survive continual TKI-exposure allows them to act as a source from which *de novo* resistance clones can arise. Ramirez et al (2016) demonstrated that diverse mechanisms of resistance could develop from DTCs-derived from a single EGFR-addicted lung cancer cell [6]. Hata et al. (2016) further demonstrated that T790M-resistant populations which evolved from DTCs were significantly less susceptible to WZ4002, a third generation EGFR TKI, when compared to populations derived from pre-existing subclones [7]. Thus, the ability of these DTCs to give rise to unpredictable, diverse, and potentially more potent mechanisms of resistance adds to the complexity of tumor heterogeneity and personalized medicine. There is an obvious need to explore DTCs further in the context of improving efficacy of TKI therapy. Ma's group has now reported their further study of this drug-tolerant population by characterizing the transcriptomic and metabolomic changes in DTCs [8].

Thiagarajan et al (2016) demonstrated large transcriptomic changes in HCC827 and H1975 cell lines after 9 days of EGFR TKI treatment, with a return to baseline expression levels after 7 days of drug “wash-out” [8]. This early onset adaptive drug-evading signature showed decreases in processes such as cell cycle regulation and proliferation, and increases in functions such as cell adhesion and adoptive stem cell signaling. Due to its presence in a significant number of these altered processes, TGFβ2 was identified as potentially having a central role in the early-adaptive response to TKI. Indeed, they demonstrated that TGFβ2 had similar protein expression patterns as phospho-STAT3 and its ability to induce the expression of the anti-apoptotic proteins BCL-2 and BCL-xL, a potential mechanism for increased drug tolerance. Further analysis of their transcriptomic data revealed decreased expression of several key genes involved in glucose metabolism (and thus the Warburg effect) after initial EGFR TKI treatment. A mass-spectrometry based global metabolomics profiling identified novel adaptive metabolomics reprogramming in the DTCs that survived TKI treatment. This reprogramming included suppressed glycolytic pathway, TCA cycle pathway, branched chain amino acids metabolism, lipid biogenesis, and enhanced inflammatory metabolism. The authors note that these findings are of significant clinical importance as this early adaptive escape response would impede detection by FDG-PET scanning, eliciting a radiographic complete response designation.

The transcriptomic and metabolomic characterization of early response to EGFR TKIs has provided further evidence for the role of BCL-2/BCL-xL signaling in early response to TKI treatment. The new link between TGFβ2 signaling with the previously reported pro-survival BCL-2 and BCL-xL signaling pathway in drug tolerance is intriguing and warrants further investigation, especially using other pre-clinical models such as patient-derived xenograft (PDX) models and clinical tissues. Their characterization of the metabolic reprogramming after TKI treatment may have identified novel therapeutic avenues.

While little more than anecdotal evidence exists to suggest a role for the DTC phenotype in the clinical development of acquired resistance, plenty of pre-clinical work is amassing. Regardless if some or all patients have pre-existing resistant subclones prior to treatment, it seems logical that this ability of cancer cells to adapt and tolerate treatment will remain an issue for future targeted therapy strategies. Thus, further characterization of all mechanisms of drug resistance and tolerance should identify optimal drug cocktails to inhibit all origins of resistance, hopefully bringing us that much closer to the cure.
